# Mass GGBFS Concrete Mixed with Recycled Aggregates as Alkali-Active Substances: Workability, Temperature History and Strength

**DOI:** 10.3390/ma16165632

**Published:** 2023-08-15

**Authors:** Yanlin Huo, Jinguang Huang, Xiaoyu Han, Huayang Sun, Tianan Liu, Jingya Zhou, Yingzi Yang

**Affiliations:** 1 School of Civil Engineering, Harbin Institute of Technology, Harbin 150090, China; 20b933061@stu.hit.edu.cn (Y.H.); hxyhitwh2015@163.com (X.H.); shy202204@163.com (H.S.); 2 Key Lab of Structures Dynamic Behavior and Control of the Ministry of Education, Harbin Institute of Technology, Harbin 150090, China; 3 Key Lab of Smart Prevention and Mitigation of Civil Engineering Disasters of the Ministry of Industry and Information Technology, Harbin Institute of Technology, Harbin 150090, China; 4 Technology and Quality Department, China MCC5 Group Co., Ltd., Chengdu 610063, China; huangjinguangtt@hotmail.com; 5 Institute of Engineering Research, Shanghai Construction No 4 Group Co., Ltd., Shanghai 200131, China; sun1085360116@163.com; 6 College of Geography and Ocean Sciences, Yanbian University, Yanji 133000, China; 0000008750@ybu.edu.cn

**Keywords:** mass concrete, recycled aggregate, ground granulated blast furnace slag (GGBFS), workability

## Abstract

This study provides the results of an experiment on the possibility of using high-volume ground granulated blast furnace slag (HVGGBFS)-based concrete as mass concrete. In addition to the control concrete, the total weight of the binder was 75% ground granulated blast furnace slag (GGBFS) and 25% ordinary Portland cement (OPC). For the aggregates, both natural and recycled aggregates were used. Three specimens with dimensions of 800 mm × 800 mm × 800 mm were prepared to simulate mass concrete. The workability, temperature aging and strength of the mass concrete were tested. The test results showed that utilizing HVGGBFS concrete as mass concrete can significantly reduce the heat of hydration due to the low heat of hydration of GGBFS, while the heat of hydration of GGBFS and recycled aggregate combination is 11.2% higher than normal concrete, with a slump that is 31.3% lower than that of plain concrete. The results also showed that the use of recycled aggregates in HVGGBFS concrete can significantly reduce workability. However, the compressive strength is higher than when natural aggregates are used due to the alkali activation effect caused by the recycled aggregates. The compressive strength at 7 and 28 days increased by 33.7% and 16.3%, respectively.

## 1. Introduction

The sustainability of concrete, the second most consumed material on the earth, is of paramount importance [1,2,3,4]. Rapid urbanization [5] and population growth [6] are factors increasing the demand for new infrastructure and housing, further exacerbating the environmental impact of the construction industry [7,8]. Global warming is currently one of the most serious environmental problems. Cement manufacture is a major factor contributing to global warming as 8% of CO_2_ emissions are due to cement manufacturing [9,10,11,12]. Therefore, the development of alternative binders is an effective solution for global warming [13,14,15,16,17,18]. Green and environmentally friendly construction can be achieved by utilizing natural resources to the least extent [19,20,21,22]. Ground granulated blast furnace slag (GGBFS) is a kind of industrial by-product generated during pig iron manufacturing [23,24,25,26]. As the chemical composition of GGBFS is similar to that of cement, many researchers focus on the use of GGBFS as an alternative binder in concrete [27,28]. Since the 1960s, a new type of alkali-activated slag concrete (AASC) using GGBFS and an alkali activator has been rapidly developed [29,30,31,32]. Many researchers found using alkali activators such as NaOH and water glass could activate the potential hydraulicity of GGBFS [33,34,35,36]. Partial replacement using GGBFS can highly reduce the environmental burden caused by cement manufacturing.

Also, the demand for construction aggregates globally in the construction industry is rising at a rate of approximately 5.2%, which reached 51.79 billion metric tons in 2019 [37,38,39,40,41]. This had led to an increasing concern connected with diminishing natural resources and environmental depletion. Approximately 850 × 10^6^ tons of solid waste generated in Europe is due to demolition activities, which is approximately 31% of the total waste generated, out of which only 3% of them are being used as aggregate originating from recycled aggregates (RA) [42,43,44]. Hence, disposal of this construction and demolition (C&D) waste is a growing concern among environmentalists. Also, using recycled concrete aggregates (RCA) for construction practices to the maximum extent wherever possible, not only reduces the harmful effects on the environment but also addresses the issue of C&D waste management [45,46].

Recycled concrete aggregates are one kind of building material obtained by crushing abolished construction waste [47,48,49]. Numerous studies have focused on the strength and durability of concrete using recycled aggregates [50,51,52]. However, it is difficult to ensure the homogeneity of the recycled aggregates. Compared with using natural aggregates, concrete using recycled aggregates may lead to defects including low workability, low strength and other durability problems [53,54,55]. Because of the low performance of recycled concrete aggregates, it is commonly believed that the replacement ratio of recycled concrete aggregates cannot be higher than 30% for the total mass of aggregates [56,57,58,59]. An important factor is the impurities that adhere to the surface of recycled concrete aggregates [60,61,62]. These impurities are mainly obtained from the cement paste of old buildings and contain large amounts of alkaline substances [63,64].

Because of the high alkaline level of the impurities, using recycled concrete aggregates as alkali activators is a possible way to activate the potential hydraulicity of the GGBFS [65]. According to the study conducted by Kathirvel, using 50% recycled coarse aggregates can obtain a higher compressive strength than 100% natural aggregates used in AASC [66]. It seems that RCA may supplement the alkali contents in the concrete and play a role during hydration [66]. The study provides useful information on the mechanical performances of GGBFS-based concrete utilizing recycled concrete aggregates. It is also considered a very efficient way to reuse recycled concrete aggregates [33,67]. However, as the study did not emphasize the alkali activate effect of RCA, further studies are needed.

Mass concrete used in many structural and non-structural applications, such as raft foundations, large size columns, concrete blocks for quay walls, suffers from cracking at early ages from the heat generated due to the hydration reaction of the binder (cement) [68]. The heat from the hydration reaction developing at the core of mass concrete element diffuses slowly to the surfaces at a rate depending on the thermal diffusivity property of concrete, resulting in a temperature increase in the core. On the other hand, at the bounding surfaces of the structural element, the generated heat is dissipated at a rapid pace to the ambient environment depending primarily on the convective heat transfer coefficient [69,70]. The heat trapped at the core of the mass concrete has two major impacts, which compromises the integrity and long-term durability of the mass concrete structures [71]. In general, the most common approaches are the use of low-heat generating binders such as fly ash, or the use of supplementary cementitious materials such as GGBFS. However, current research is more focused on the incorporation of fly ash in mass concrete.

Malhorta and Mehta studied the effects of high-volume fly ash (HVFA) concrete, with fly ash making up more than 50% of the total mass of the binder, and obtained even better durability performances than OPC-based concrete [41,72,73,74]. These attributes are extremely beneficial when used as mass concrete [75,76,77]. Similar to fly ash, GGBFS could also reduce hydration heat in concrete [78,79]. However, very few studies show the performances of HVGGBFS mass concrete [78,80,81,82,83]. Current research on high volume GGBFS is focused on optimizing early age cracking (EAC) and shrinkage. For example, Shen et al. [84] conducted restrained ring tests on GGBFS concrete with GGBFS content varying from 0 to 50% and also found that the increasing content of GGBFS decreased the EAC risk. Under semi-adiabatic conditions, Wei et al. [85] carried out uniaxial restrained tests and concluded that, although the thermal shrinkage can be effectively reduced, the major reasons accounting for EAC of GGBFS concrete lay in the consistent autogenous shrinkage. Focusing on the influence of slag composition, Markandeya et al. [86] conducted TSTM tests on GGBFS concrete using GGBFS with different MgO/Al_2_O_3_ ratios, but with similar Ca/SiO_2_ ratios. Their results showed that a low MgO/Al_2_O_3_ ratio can result in high autogenous shrinkage and therefore promote the EAC risk. However, these studies have not deeply explored the contribution of GGBFS to the heat of hydration in mass concrete. In addition, besides GGBFS, a variety of inexpensive wastes are needed to enter the concrete system. Recycled aggregates are a good alternative. The old mortar on its surface can assist the hydration of GGBFS to occur.

To summarize, it is very necessary to go for introducing high-volume GGBFS and recycled aggregates into the concrete system for economic and ecological benefits. The alkaline nature of the waste mortar on the surface of recycled aggregate and the potential hydration properties of GGBFS is utilized to alleviate the problem of exothermic cracking inside the mass concrete and to expand new ideas for the development of concrete.

This study focuses on the possibility of mass concrete with HVGGBFS and the alkali activation after RCA incorporation. Based on this, mock-up tests of mass concrete with HVGGBFS as a binder are provided. Workability, compressive strength and temperature age are tested to compare the different binders. To examine the effect of recycled aggregates as an alkali activator, both recycled and natural aggregates are used in HVGGBFS concrete. This study also developed a strategy for producing mass concrete with lower internal temperature.

## 2. Experimental Scheme

### 2.1. Materials

In this study, P·O42.5 ordinary Portland cement (OPC) from Yatai Group Harbin Cement Company (Harbin, China) and the S95 grade ground granulated blast furnace slag (GGBFS) from a local processing plant (Harbin, China) were used as binders, and the density was 3.15 g/cm^3^ and 2.90 g/cm^3^, respectively. The chemical composition of OPC and GGBFS is given in Table 1. According to the market survey, the costs of OPC and GGBFS are CNY 450 per ton and CNY 250 per ton, respectively. GGBFS has obvious economic benefits.

**Figure 1:**
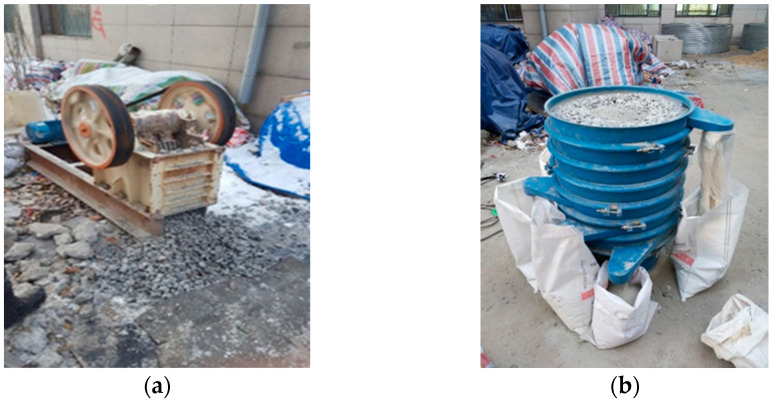
Preparation of RCA and RFA. (**a**) Crushing concrete with a jaw crusher. (**b**) Separation of RCA and RFA.

**Figure 2:**
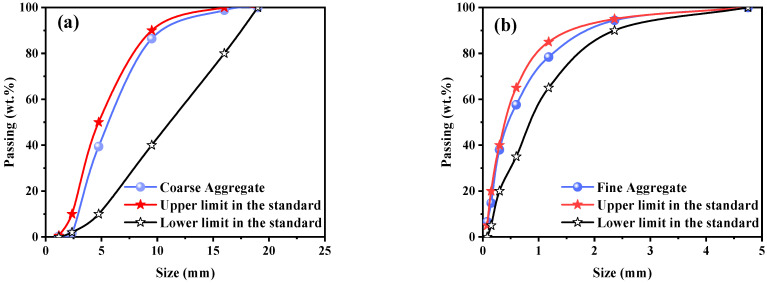
Distribution of aggregate particle size: (**a**) coarse aggregate and (**b**) fine aggregate.

A total of four types of aggregates were selected for use: natural coarse aggregate (NCA), natural fine aggregate (NFA), recycled coarse aggregate (RCA), and recycled fine aggregate (RFA), respectively. The natural aggregates are gravel and river sand, which come from the local quarries in Harbin. The recycled aggregates came from a local construction site in Harbin, where the waste concrete was crushed by a jaw crusher, as shown in Figure 1. The chemical composition and physical properties of the aggregates are listed in Table 2. The recycled aggregates used the same particle gradation as the natural aggregates to control the consistency of the test conditions. The particle gradations of coarse and fine aggregates are given in Figure 2, respectively.

The proportions of the mass concrete are given in Table 3, where the binder for the control group was 100% OPC, while the other two groups replaced an equal mass of OPC with 75% GGBFS. The most common water–binder ratio is 0.5, the binder–sand ratio is 0.46, and the percentage of sand in aggregates is 0.43. According to the difference between binders and aggregates, the concrete mixtures are named ONN (OPC − NCA − NFA), BNN (OPC + GGBFS − NCA − NFA) and BRR (OPC + GGBFS − RCA − RFA).

### 2.2. Proportion of Concrete

**Figure 3:**
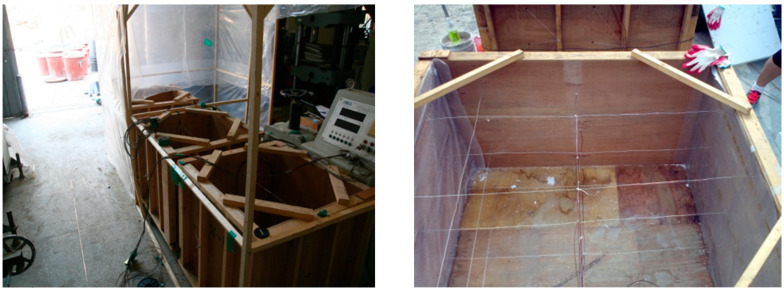
Mould preparations for mass concrete.

Three days before the preparation of concrete, the coarse and fine aggregates are dried in a natural environment to a dry condition (moisture content approximated to 0). One hour before preparation, the RCA and RFA were pre-absorbed and the amount of water absorbed was calculated based on the water absorption of the recycled aggregate over the natural aggregate. Four 150-L capacity concrete mixers were used to prepare the mass concrete together (one mass concrete specimen was approximately 520 L). The solid components, including OPC, GGBFS, and coarse and fine aggregates, were first mixed for 2 min. Water was then added to the mixer and mixing continued for 2 min. All the fresh concrete mixture was poured into the mould (800 mm × 800 mm × 800 mm) as shown in Figure 3 and pounded with a pounding bar. Finally, the surface was smoothed and cling film was attached, and 72 h later, the wooden formwork was removed and the concrete specimens were watered and cured.

### 2.3. Testing Procedures

#### 2.3.1. Fresh Properties Tests

The slump, air content and chloride contents of fresh concrete were tested immediately in compliance with test methods regulated in ASTM C143/143M [87], C231 [88] and JGJ/T 322-2013 [89], respectively.

#### 2.3.2. Temperature History

**Figure 4:**
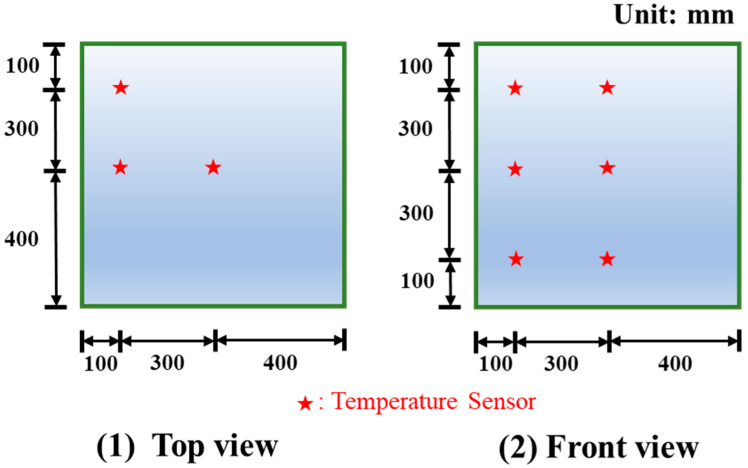
Schematic diagram of the arrangement of temperature sensors in mass concrete.

**Figure 5:**
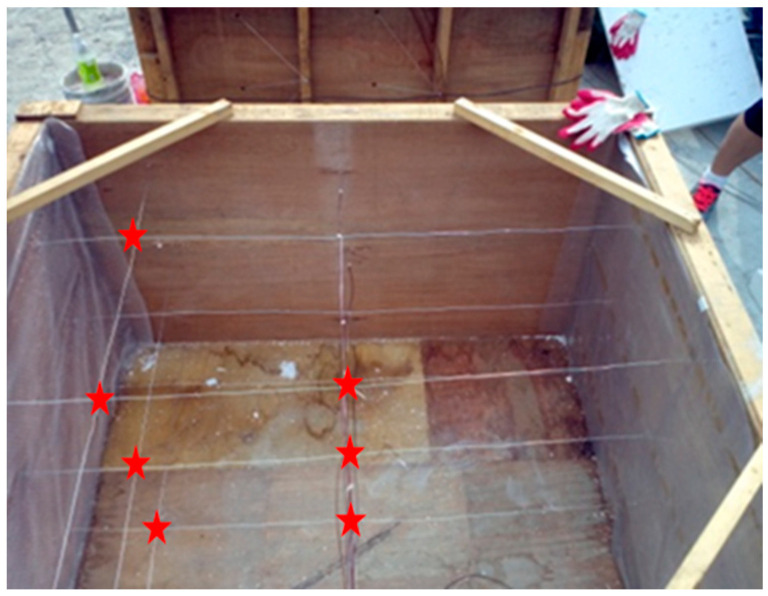
Actual diagram of the arrangement of temperature sensors in mass concrete. The red star represents the temperature sensor.

The authors conducted detailed field monitoring of temperature rise in mass concrete blocks. As shown in Figure 4 and Figure 5, nine temperature sensors were installed at three different locations in the centre, corners and edges of the concrete at the top, middle height and bottom. Temperature changes were measured at 10 min intervals over 10 days.

#### 2.3.3. Compressive Strength Test

The compressive strengths of concrete mixes were determined at 3, 7 and 28 days of age. Concrete cylinders of 300 mm × 150 mm were used for the compressive strength tests as per ASTM C39 [90]. The loading rate was kept at 0.2 MPa/s. An average of three specimens was used to perform the test. A compression testing machine with a capacity of 2000 kN was used for these tests.

#### 2.3.4. Rebound Number

The rebound number test of hardened concrete was conducted according to the methods specified in ASTM C805/805M [91]. All test pictures can be seen in the Support Information.

#### 2.3.5. Micro-Analysis

Samples at the age of 7 and 28 days were immersed into isopropanol for one week to stop the reaction and dried in a 40 °C oven for 1 h for the SEM studies. The surface of the samples was coated by carbon and subsequently dried in a low vacuum desiccator before analysis. Afterwards, the fractured and polished samples were observed by SEM with the secondary electron (SE) mode at an acceleration voltage of 20.0 kV under a low vacuum, respectively.

## 3. Results and Discussion

### 3.1. Fresh Properties

**Figure 6:**
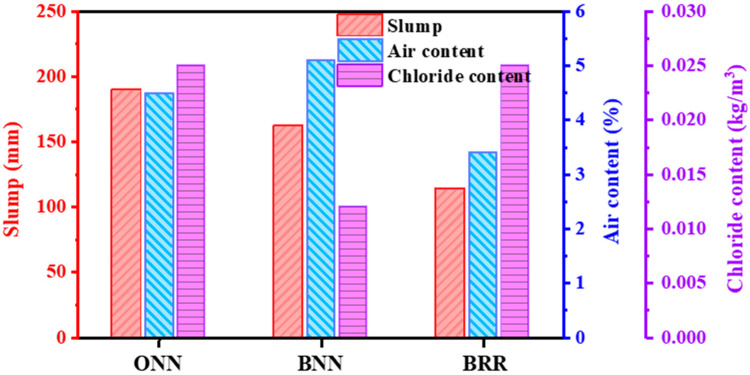
Slump, air content, and chloride content of concrete.

Figure 6 shows the results of the slump, air content and chloride content depending on the mixture combination. The slump result of ONN is 190 mm, which is higher than BNN and BRR. The higher water absorption of GGBFS and the alkali activation of recycled aggregates are considered to be the main reasons for the lower slump of BNN and BRR [33]. If too much GGBFS is added (up to 75% in this paper), the water requirement is too large and the viscosity of the slurry is unfavourable for flow.

The air content ranges from 3.4% to 5.1%, which satisfied the target range. The high water absorption of RCA leads to a decrease in the effective water–cement ratio, which in turn introduces more air pockets and increases the air content of the material. Moreover, there are micro-cracks and voids in the paste attached to the surface of RCA, which also leads to an increase in the air content.

The three types show a lower than 0.30 kg/m^3^ chloride content, which satisfies the standard range [89]. When a high volume of GGBFS is used, the content of chloride ions within the concrete decreases significantly, which is very much utilized for the safety of reinforced concrete structures. It is well known that corrosion of reinforcing bars in reinforced concrete is a very serious problem, and the most important reason for this lies in the erosion of chloride ions. Therefore, the incorporation of large quantities of GGBFS is helpful in the protection of steel reinforcement. Also, it was found that since recycled aggregates contain old mortar, they have a higher chloride ion content and introduce twice as many chloride ions as natural aggregates for the same mix ratio. Therefore, the subsequent application of recycled aggregates requires strict monitoring of the chloride ion content within the concrete.

### 3.2. Temperature History

**Figure 7:**
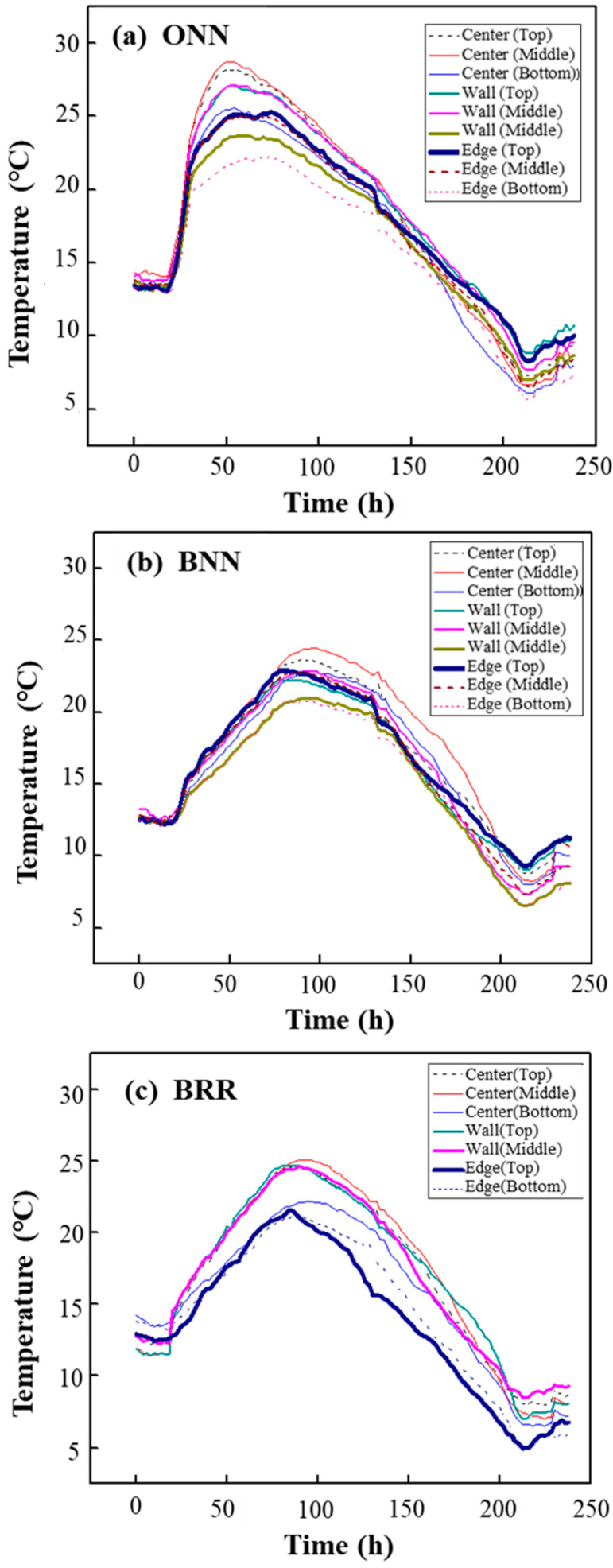
Temperature history with age: (**a**) ONN, (**b**) BNN and (**c**) BRR.

Figure 7 shows the temperature history of the specimens with age. After pouring, the temperature of all specimens went up gradually. For the ONN specimen, 50 h after placing, the highest temperature of 28.7 °C appeared in the centre position of the specimen, and the temperature at the edge of the specimen was 23 °C, which makes a temperature difference of 5.7 °C between the centre and the edge. For the BNN specimen, it took about 100 h to reach the peak temperature, which is about two times later than the ONN specimen. The highest temperature of BNN is 24.5 °C, which is 3 °C lower than ONN. This is due to the larger OPC content in the ONN mixture than in the BNN mixture. The BRR specimen reached the peak temperature faster than BNN, and the highest temperature was 1.5 °C higher than the BNN specimen. This may be because the alkalinity of recycled aggregate in BRR played a role in activating the potential hydraulic of GGBFS, thus it contributed to raising the temperature of BRR [33].

Previous studies have shown that the risk of thermal cracking in large concrete infrastructure is highly dependent on the temperature development and the maximum temperature [92]. The results of this study show that concrete containing GGBFS has the potential to reduce the risk of cracking in structures such as dams and tunnels.

The temperature change is closely related to the performance of the material in terms of the heat of hydration. Gao et al. [93] tested the heat of hydration of GGBFS slurries with substitution rates ranging from 0 to 40% and found that the heat of hydration of the slurries decreased significantly with the increase in the dosage of GGBFS. The reason for this is the low hydration activity of GGBFS in the early stage, which leads to the decrease in hydration products in the first 72 h.

Considering the limitations of the heat of hydration test, we could not measure the effect of RCA on the heat of hydration of HVGGBFS concrete. Given this, since recycled fine aggregate (RFA) and RCA have similar compositions, we tested the effect of RFA on the heat of hydration, details of which can be found in [33]. RFA increases the heat of the solution due to the higher amount of CaO. CaO reacted with water to form Ca(OH)_2_ and released heat. It is considered the reason that a higher peak is observed in RFA-based specimens. Similarly, the old mortar attached to the surface of the RCA had a large amount of residual cement. These cement particles with a high quantity of CaO increase the alkali concentration and further activate the hydraulicity of GGBFS.

### 3.3. Compressive Strength

**Figure 8:**
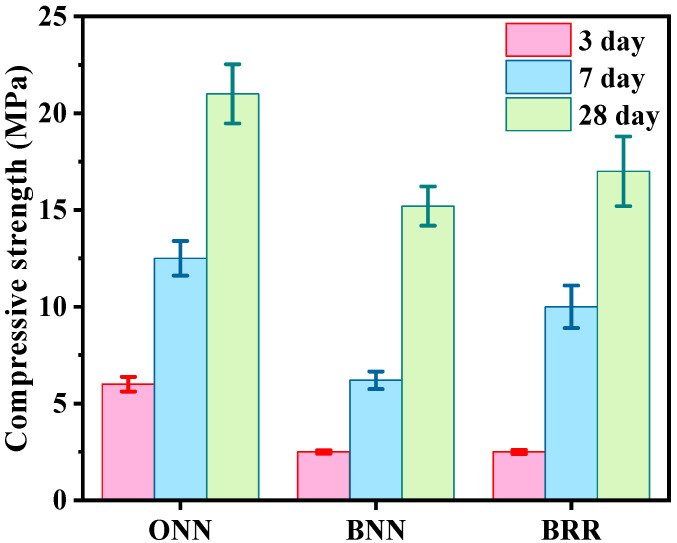
Compressive strength with different mixture.

Figure 8 shows the 3-, 7- and 28-day compressive strength of each specimen. ONN had a higher compressive strength than BNN; 53.2%, 48.6% and 26.3% higher at 3, 7, 28 days, respectively. Hydration of concrete slowdowns when GGBFS is added to it, thus leading to a lower hydration of heat. It is due to the reason that GGBFS reacts slowly to form a C-S-H gel and most of its content reacts with calcium hydroxide to form a C-S-H gel and thus a delay in hydration. Early strength whether compressive or flexural both reduced due to slow hydration but with increased curing period strength both compressive and flexural are increased considerably due to further formation of C-S-H gel by GGBFS. Due to slow hydration, the early strength of concrete is compromised and thus needs more supervision in its initial stages while, as provided with a longer curing period, GGBFS tends to produce concrete showing much better results in terms of strength in the long run.

ONN has 54.7%, 22.4% and 12% higher strength than BRR at 3, 7, 28 days as expected. According to the literature, the use of 100% RFA in OPC-based concrete leads to a loss of compressive strength ranging from 39% to 60% [94,95]. In comparison, the use of RFA in HVGGBFS concrete is a more efficient method of use. When comparing BNN with BRR, they showed similar strength at 3 days, while BRR has 33.7% and 16.3% higher strength than BNN at 7 and 28 days, respectively. This can be explained as the mortar from old buildings adhered to the surface of recycled aggregates with high alkalinity; the potential hydraulic of GGBFS is activated by the high alkalinity from the recycled aggregates.

### 3.4. Rebound Number

**Figure 9:**
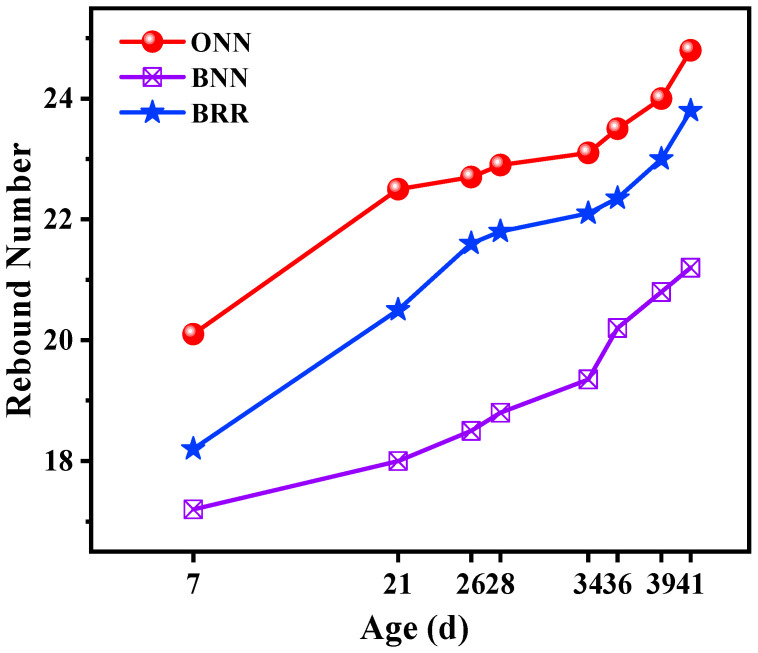
Rebound number of different mixtures with age.

Figure 9 shows the specimens’ rebound number with age. ONN, which has the highest compressive strength, has the highest rebound number. BRR has a higher rebound number than BNN. The reason is the same as the reason for the compressive strength mentioned before.

### 3.5. Micro-Analysis

**Figure 10:**
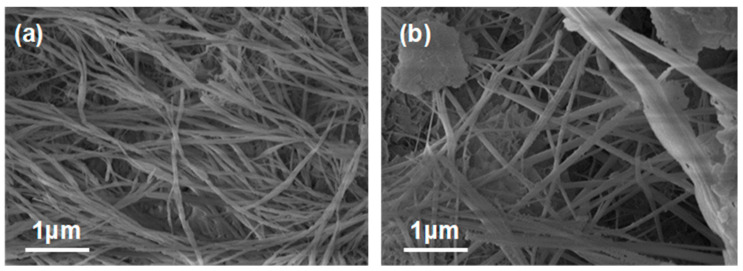
SEM images of BRR: (**a**) 7 days and (**b**) 28 days.

Figure 10 shows the SEM images of BRR at 7 and 28 days. The images both indicate fine and long needle-like structures, i.e., Ettringite. Ettringite has a very low solubility product among all hydration products and is very susceptible to precipitation crystallization, exhibiting an impact on early mobility. Simultaneously, the formation of Ettringite can promote the early strength development of cement, and the mechanical occlusion between the radiolucent calcium alumina crystal clusters provides significant early strength. Additionally, for large-volume cement concrete projects or high-temperature cured concrete products, the calcium alumina formed will decompose due to the high internal temperature in the early stage, and Ettringite will be formed again in the late stage of cement hardening, which will easily cause the cracking and damage of concrete. As the amount of GGBFS is larger, it significantly reduces the internal exotherm of large-volume concrete.

## 4. Conclusions

In this study, the potential for the incorporation of high volumes of GGBFS and recycled aggregate into a mass concrete system at the same time was emphasized. Slump, air content and chloride content of fresh concrete were tested. And three 800 mm × 800 mm × 800 mm mass cube specimens were tested for monitoring the internal heat of hydration and rebound number. Also, the compressive strength of concrete was tested at 3, 7, and 28 days. The main conclusions obtained are as follows:

(1) For fresh concrete, slump decreases with increasing GGBFS content and aggregate dosage; 75% GGBFS and recycled aggregate combination gives a 31.3% lower slump than BNN with natural aggregates. The chloride content of all specimens was less than 0.3 kg/m^3^.
(2) The higher volume level of GGBFS replacement resulted in a significant reduction in heat of hydration compared to OPC. The combination of GGBFS and recycled aggregate showed 11.2% higher heat of hydration than BNN of natural aggregate.
(3) At 28 days of age, high-volume GGBFS reduces compressive strength compared to ONN. The compressive strength at 7 days and 28 days with recycled aggregate was 33.7% and 16.3% higher than with natural aggregate when high-volume GGBFS was used as a binder.

## Figures and Tables

**Table 1:** The chemical composition of OPC and GGBFS (wt. %).

Ingredients	SiO_2_	Al_2_O_3_	Fe_2_O_3_	CaO	MgO	Others
OPC	21.4	5.45	3.5	64.48	1.46	3.71
GGBFS	27.6	13.51	0.54	42.96	9.31	6.08

**Table 2:** The chemical composition and physical properties of the aggregate.

Aggregate	Physical Properties			Chemical Composition (wt. %)							
	Fineness Modulus	Water Absorption (%)	Density (g/cm^3^)	SiO_2_	CaO	Al_2_O_3_	Na_2_O	K_2_O	MgO	Cl^−^	Others
RCA	6.14	4.20	2.65	73.62	9.12	7.48	0.96	1.17	3.58	0.74	3.33
RFA	2.76	6.20	2.58	60.40	16.96	10.65	1.87	3.10	1.00	0.13	5.89
NCA	6.48	0.58	2.76	96.71	0.23	0.63	0.08	0.14	0.24	0.01	1.96
NFA	2.86	2.63	2.69	75.59	1.39	13.18	3.28	4.42	0.11	0.02	2.01

**Table 3:** Mixture proportion of concrete (kg/m^3^).

Mixture ID	OPC	GGBFS	Coarse Aggregate		Fine Aggregate		Water
			Gravel	Recycled	River Sand	Recycled	
ONN	360	0	1027	-	775	-	180
BNN	90	270	1027		775		180
BRR	90	270	-	1000	-	755	180

## Data Availability

Data sharing not applicable.

## Supplementary Materials

The following supporting information can be downloaded at: https://www.mdpi.com/article/10.3390/ma16165632/s1.
