# Supplementary material for: Mass GGBFS Concrete Mixed with Recycled Aggregates as Alkali-Active Substances: Workability, Temperature History and Strength
Source: Materials (Basel). 2023 Aug 15;16(16):5632. doi: 10.3390/ma16165632 (PMC10456633; doi:10.3390/ma16165632)
Supplement: Supplementary file 1 [file materials-16-05632-s001.zip › materials-2530739-supplementary.pdf]

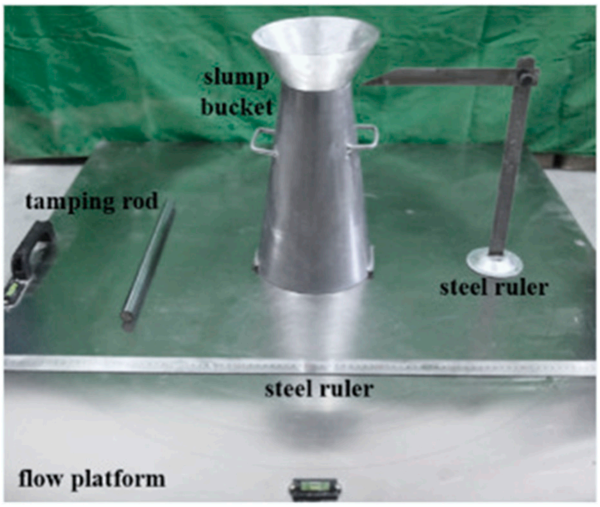

Slump test

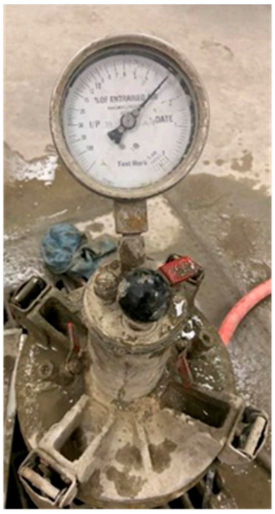

Air content test

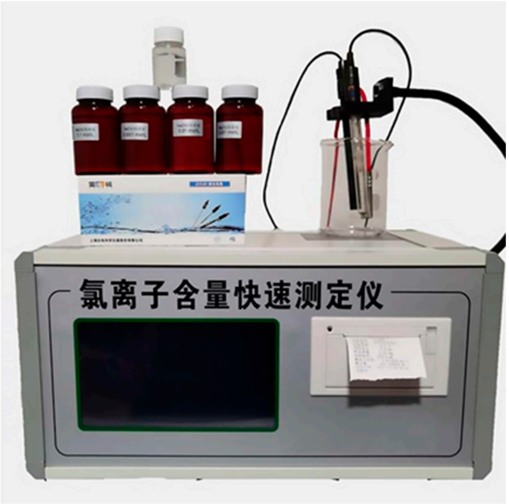

Chloride content test

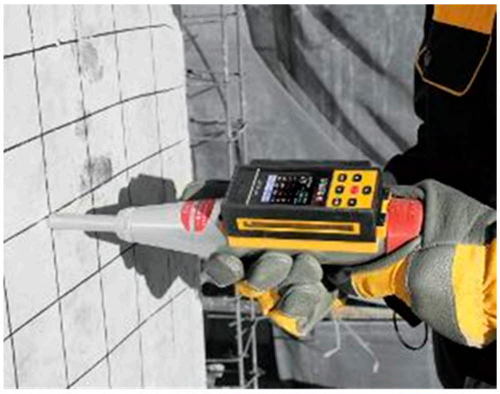

Rebound number test

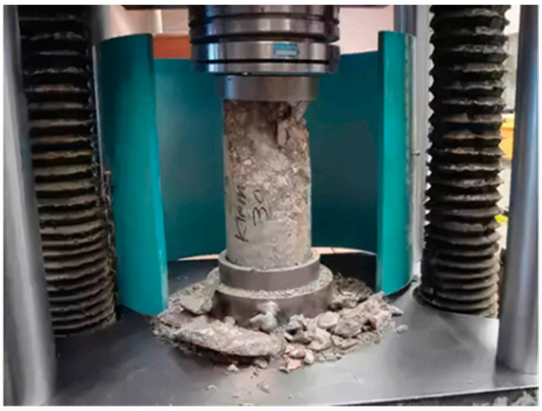

Compressive strength test
